# Medical Treatment of Advanced Pancreatic Neuroendocrine Neoplasms

**DOI:** 10.3390/jcm9061860

**Published:** 2020-06-15

**Authors:** Lola-Jade Palmieri, Solène Dermine, Amélie Barré, Marion Dhooge, Catherine Brezault, Anne-Ségolène Cottereau, Romain Coriat

**Affiliations:** 1 Gastroenterology and Digestive Oncology Department, Cochin Hospital, 75014 Paris, France; solene.dermine@aphp.fr (S.D.); amelie.barre@aphp.fr (A.B.); marion.dhooge@aphp.fr (M.D.); catherine.brezault@aphp.fr (C.B.); romain.coriat@aphp.fr (R.C.); 2 Faculty of Medicine Paris Centre, University of Paris, 75006 Paris, France; annesegolene.cottereau@aphp.fr; 3 Nuclear Medicine Department, Cochin Hospital, 75014 Paris, France

**Keywords:** pancreatic neuroendocrine tumor, pancreatic neuroendocrine neoplasm, pancreatic neuroendocrine carcinoma, neoadjuvant, palliative, chemotherapy, targeted therapy

## Abstract

Pancreatic neuroendocrine neoplasms (panNENs) are relatively rare but their incidence has increased almost sevenfold over the last four decades. Neuroendocrine neoplasms are classified according to their histologic differentiation and their grade. Their grade is based on their Ki-67 proliferation index and mitotic index. Their prognosis is highly variable according to these elements and treatments also vary according to their classification. Surgery is the only curative treatment for localized and advanced panNENs and offers a better prognosis than non-surgical treatments. In the case of an advanced panNEN without the possibility of resection and/or ablation, medical treatment remains the cornerstone for improving survival and preserving quality-of-life. PanNENs are considered as chemosensitive tumors, unlike midgut neuroendocrine tumors. Thus, panNENs can be treated with chemotherapy, but targeted therapies and somatostatin analogs are also treatment options. The scarcity and heterogeneity of NENs make their management difficult. The present review aims to clarify the medical treatments currently available for advanced panNENs, based on their characteristics, and to propose a treatment algorithm.

## 1. Introduction

Pancreatic neuroendocrine neoplasms (PanNENs) are considered as relatively rare neoplasms. However, their incidence has increased almost sevenfold over the last four decades: the annual age-adjusted incidence of neuroendocrine neoplasms (NENs) was 1.09 per 100,000 people in 1973 versus 6.98 per 100,000 people in 2012 [1]. This increase in incidence may be related to a better knowledge of NENs by physicians but also to better diagnostic tests. PanNENs are less frequent than those of the small intestine or rectum [1]. Their prognosis is highly variable and depends in particular on histological differentiation, Ki-67 proliferation index, and tumor stage.

The World Health Organization (WHO) classifies NENs as well-differentiated or poorly-differentiated. They are also classified as grade 1 (G1), grade 2 (G2) and grade 3 (G3) on the basis of the Ki-67 index or mitotic index. Well-differentiated NENs are defined as neuroendocrine tumors (NETs) and poorly differentiated G3 NENs are defined as neuroendocrine carcinomas (NECs) [2]. G3 NETs and NECs are treated differently from G1 and G2 NETs. Pancreatic mixed neuroendocrine non-neuroendocrine neoplasms (MiNENs) are a mixture of mixed carcinomas with panNENs.

Surgery is the only curative treatment for localized and metastatic panNENs and offers a better prognosis than non-surgical treatments [3]. However, if the patient has a metastatic panNEN without the possibility of curative surgical treatment or locoregional or ablative therapy, or if there is a high surgical risk, medical treatment remains the cornerstone for improving survival and preserving quality-of-life. Unlike in well-differentiated midgut NETs, where the cytotoxic chemotherapy is thought to have limited activity, panNENs are considered as chemosensitive tumors. Among recently approved therapies, a temodal + capecitabine (TEMCAP) regimen has been recommended for the management of panNENs, with improved cytoreduction and prolonged survival. Certain targeted therapies such as everolimus and sunitinib have also been shown to be effective in panNENs. Thus, the survival of panNENs has steadily increased in the last three decades, reflecting improved therapies [1]. Treatment algorithms are regularly reviewed by the European NeuroEndocrine Tumor Society (ENETS) and North American NeuroEndocrine Tumors Society (NANETS), but some challenges remain, including predictive biomarkers of response and appropriate combinations of therapies.

The scarcity and heterogeneity of NENs render their management difficult and explains the low number of randomized studies and the low level of evidence associated with them. Thus, algorithms and treatment combinations can be argued and it is recommended to discuss NENs cases in a dedicated and specialized multidisciplinary tumor board. Given the impact of pathology findings in NENs, it is also recommended to have the pathology slides reviewed by a specialized pathologist.

This review focuses on the medical treatment of metastatic panNENs, excluding surgical, locoregional, and ablative therapies. The different therapeutic options for well-differentiated G1 or G2 pancreatic neuroendocrine tumors (panNETs) and for G3 panNENs will be detailed, along with recent data.

## 2. Well-Differentiated G1/G2 PanNETs

Medical treatments should be considered for well-differentiated G1/2 panNETs, if no curative surgical or locoregional or ablative therapy is possible. Surgical resection and/or destruction may be considered in advanced panNETs in the case of macroscopically resectable liver metastases, with no or little disease progression after several months of follow-up or systemic treatment. Resection and/or destruction appear to be associated with a longer survival, although it has never been properly compared with other treatments. Conversely, surgical resection is not possible in the case of a rapidly progressing panNET, or with significant or unresectable hepatic invasion, or in the case of multiple metastases.

### 2.1. Somatostatin Analogs (SSAs)

Pancreatic islet-cell tumors and gastrointestinal NETs retain many characteristics of the neuroendocrine cells from which they arise, and more than 80% of well-differentiated NETs express somatostatin receptors (SSTRs) [4]. SSAs will bind with a high affinity to SSTRs.

Symptoms from hormonal hypersecretion are common in functional metastatic panNETs and symptom control is often urgent. Octreotide and lanreotide are long-acting SSAs. They are considered equally effective for symptom control [5] and are approved for antisecretory therapy.

SSAs also have anti-proliferative activity. In the PROMID phase III trial, 85 patients with well-differentiated functional and nonfunctional metastatic midgut NETs were randomized to receive 30 mg of long-acting release (LAR) octreotide or placebo monthly. Octreotide significantly improved the median time to tumor progression (mTTP) compared to placebo: 14.3 versus 6 months, respectively (hazard ratio (HR) 0.34, *p* < 0.001) [6]. This trial demonstrated the efficacy of SSAs for midgut NETs but excluded panNETs. Later, the phase III CLARINET trial randomized 204 patients with advanced well-differentiated and non-functional G1 or G2 panNETs to receive lanreotide autogel 120 mg or placebo monthly [7]. At 24 months, mPFS was not achieved for lanreotide versus 18 months for placebo (HR 0.47; *p* < 0.001). An extension of the CLARINET study estimated the median progression-free survival (mPFS) of lanreotide to be 32.8 months [7]. Lanreotide was also effective in the CLARINET study in case of a high hepatic tumor burden (>25% liver involvement). According to ENETS guidelines [8], lanreotide should be preferred to octreotide for panNETs. SSA anti-proliferative activity is probably a class effect but octreotide was never tested prospectively in panNETs, nor was it compared to lanreotide.

SSAs have a low anti-tumor efficacy with low cytoreduction rates but allow stabilization of the disease. In case of SSTR avidity, SSAs (octreotide LAR 30 mg and lanreotide autogel 120 mg every 28 days) can be used as first-line treatment of advanced panNETs with stable or slowly progressing disease, or in patients with unknown tumor behavior with Ki-67 less than 5–10%.

Patients with well-differentiated panNETs who are progressing under standard doses of SSAs may benefit from a shortened SSAs administration regimen, with a longer demonstrated mTTP [9].

Tachyphylaxis may occur in some patients with NETs treated with SSAs. This desensitization can be overcome by increasing the dose of SSAs. The most common side effects are injection site pain, abdominal pain with diarrhea, nausea and vomiting.

### 2.2. Chemotherapy

Chemotherapy is the first-line therapeutic standard for metastatic and progressive panNETs with a goal of cytoreduction.

#### 2.2.1. Alkylating Agents

Alkylating agents studied in advanced panNETs include streptozotocin, dacarbazine and temozolomide. Alkylating agents are the cornerstone of the chemotherapy regimen and are often proposed as second-line treatment after disease progression under SSAs.

**Streptozotocin** is an alkylating agent that is selectively toxic to beta cells of the pancreas [10]. Its use in panNETs was first described in a case report with a relief of hormonal symptoms as well as a cytostatic control in an insulinoma patient treated with streptozotocin [11]. Moertel et al. studied 105 patients with panNETs, randomized between streptozotocin + 5-fluorouracil, streptozotocin + doxorubicin or chlorozotocin alone. The combination of streptozotocin + doxorubicin had a significant advantage in terms of objective response rate (ORR) and survival over streptozotocin + 5-fluorouracil (ORR: 69% vs. 45%, *p* = 0.05; median overall survival [mOS] 2.2 vs. 1.4 years; *p* = 0.004) [12]. This high ORR with streptozotocin was not reproducible later in retrospective series [13,14,15,16]. In a retrospective study on 110 patients with metastatic panNETs, Ki-67 > 5% was the only predictive marker of an objective response with streptozotocin [16]. Streptozotocin renal toxicity does not appear to be a major cause of treatment discontinuation [17]. Doxorubicin causes cumulative cardiotoxicity, has a high emetogenic potential and is alopecic. These adverse effects limit doxorubicin use compared to other available combinations.

The alkylating agent **dacarbazine** has been evaluated as an alternative to streptozotocin to find a less toxic drug. Dacarbazine was tested as monotherapy in a phase II study of 50 patients with panNETs, with an ORR of 34% and a mOS of 19.3 months [18]. Dacarbazine can be used in combination with 5-fluorouracil [19]. The most common toxicities of dacarbazine are gastrointestinal (nausea/vomiting) and hematological.

**Temozolomide** is an oral alkylating agent, a prodrug of dacarbazine, used in the treatment of glioblastoma and melanoma with mild side effects. Its main toxicity is myelosuppression, particularly thrombocytopenia. Temozolomide was first studied as monotherapy in a retrospective series of 36 patients with NETs with a mean of 2.4 previous lines: the mTTP was seven months and ORR was seen in 14% of patients [20]. Temozolomide was also studied in combination with capecitabine (TEMCAP), first in a retrospective study on 18 patients progressing after SSAs and chemoembolization, with an interesting ORR of 61% [21]. TEMCAP was then studied in a phase II study on 30 patients with untreated and well-differentiated metastatic panNETs, with a 30% ORR, an 18-month PFS and a two-year survival of 92% [22]. Temozolomide was recently compared to TEMCAP in a randomized phase II on 144 patients with advanced panNETs. Preliminary results showed that TEMCAP resulted in a significant improvement in mPFS (22.7 versus 14.4 months, HR 0.58, *p* = 0.023) and mOS (not reached versus 38 months, HR 0.41; *p* = 0.012) compared to temozolomide, but no difference on ORR [23]. Conversely, a retrospective analysis on 138 panNETs patients who received temozolomide or TEMCAP showed no benefit of TEMCAP on survival but a benefit on ORR [24].

#### 2.2.2. MGMT Status

Alkylating agents induce methylation of the O^6^-position of guanine, which leads to DNA mismatching and causes apoptosis and tumor cell death. O^6^-methylguanine DNA methyltransferase (MGMT) is a DNA repair enzyme that specifically removes the methyl/alkyl group from the O6-position of guanine. Kulke et al. reported that MGMT deficiency was associated with the response to temozolomide in patients with NETs and suggested that the MGMT status could be used as a predictive marker of response to treatment with alkylating agents [25]. Retrospective series have subsequently confirmed these data [26,27]. In the absence of prospective data on a larger number of patients, routine determination of MGMT status prior to the introduction of an alkylating agent cannot be recommended.

#### 2.2.3. Platinum Agents

The efficacy of cisplatin appears to be limited to patients with NETs G3. In a prospective study on 27 patients with well-differentiated metastatic NETs treated with cisplatin + etoposide, only two patients had an objective response [28].

Oxaliplatin-based regimens have a greater activity in advanced panNETs. Two retrospective studies evaluated the capecitabine + oxaliplatin (CAPOX) and 5-fluorouracil + oxaliplatin (FOLFOX) regimen for well-differentiated NETs, with an ORR between 26% and 30% and a disease control rate (DCR) between 78% and 80% [29,30]. Two prospective phase II studies examined the efficacy of FOLFOX or CAPOX plus bevacizumab in patients with advanced NETs. Pooled data showed radiographic responses and prolonged disease stability. The 12 patients treated with FOLFOX + bevacizumab had an ORR of 41.7% and a mPFS of 21 months. The 40 patients treated with CAPOX plus bevacizumab had an ORR of 18% and a mPFS of 16.7 months [31]. The GEMOX regimen was also tested in a retrospective study of 104 patients with metastatic NETs (37 panNETs), with a mPFS of 7.8 months and a mOS of 31.6 months, and is also an alternative.

**Figure 1:**
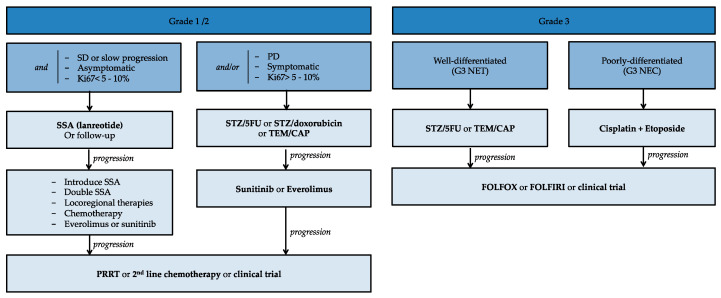
Algorithm for treating advanced pancreatic neuroendocrine tumors. 5FU: 5-fluorouracil; CAP: capecitabine; NEC: neuroendocrine carcinoma; NET: neuroendocrine tumor; PRRT: peptide receptor radionuclide therapy; SD: stable disease; SSA: somatostatin analogs; STZ: streptozotocin; TEM: temodal.

Systemic chemotherapy should be considered in G1/2 panNETs progressing rapidly in less than 6 to 12 months, or in a symptomatic patient. It should also be considered in patients without prior progression but with a high tumor burden and in patients with a chance of achieving a response to allow subsequent surgery (Figure 1).

### 2.3. Targeted Therapies

**Sunitinib** is a tyrosine kinase inhibitor (TKI) that inhibits vascular endothelial growth factor receptor (VEGFR) 1, 2, 3, platelet-derived growth factor receptor (PDGFR), colony-stimulating factor 1 receptor (CSFR) and c-KIT. Its efficacy has been demonstrated in a randomized phase III trial in patients with advanced and well-differentiated panNETs [32]. One hundred and seventy-one patients were randomized to receive either sunitinib or placebo. The study was prematurely terminated due to serious adverse events and deaths in the placebo group and a difference in PFS in favor of sunitinib. The mPFS was 11.4 months in the sunitinib group versus 5.5 months in the placebo group (HR 0.42; *p* < 0.001). The ORR was 9.3% in the sunitinib group versus 0% in the placebo group. A retrospective analysis recently confirmed these results on 171 patients receiving sunitinib or placebo [33]: sunitinib was superior to placebo on mPFS: 12.6 versus 5.8 months (HR, 0.32; *p* = 0.000015) and mOS: 38.6 versus 29.1 months (HR, 0.73; *p* = 0.094), with 69% of placebo patients crossing over to sunitinib. The standard dose of sunitinib is 37.5 mg/day. The most common adverse events of sunitinib are neutropenia, diarrhea and leukopenia [34]. Patients may also develop hypertension and foot-hands syndrome. Monitoring of sunitinib plasma concentrations may help prevent serious acute toxicities and detect patients with suboptimal exposure at the time of disease progression [35]. As the disease progresses, if underexposure is identified, the dose of sunitinib may be increased to 50 mg daily (4 weeks out of 6) [36]. Additionally, pneumatosis intestinalis has been described in patients receiving sunitinib with long-term exposure (>4 months) [37].

**Everolimus** is a rapamycin derivative that selectively inhibits mammalian target of rapamycin complex 1 (mTORC1), a key protein kinase complex that regulates cell growth, proliferation, and survival. Activation of mTORC1 is mediated by the PI3K pathway through activation of AKT/PKB and subsequent inhibition of the tuberous sclerosis complex 1/2. In the phase III RADIANT-3 study, 410 patients with advanced, G1 and G2 panNETs were randomized to receive everolimus or placebo [38]. Everolimus showed a significant benefit on mPFS: 11.0 versus 4.6 months with placebo (HR 0.35, *p* < 0.001) and on mOS: 44 versus 37.7 months for placebo (HR 0.94; *p* = 0.30), with 85% patients in the placebo arm crossover to everolimus [39]. The standard dose for everolimus is 10 mg/day. Common side effects of everolimus are asthenia, oral mucositis, digestive disorders (diarrhea, nausea), anemia, hyperglycemia and pneumonia.

**Pazopanib** is a TKI with activity against VEGFR 2–3, PDGFR and c-KIT. Pazopanib was recently evaluated in a randomized phase II placebo-controlled trial in 171 cases of progressive carcinoid tumors, and in 33% of panNET cases. Pazopanib was significantly superior to placebo in mPFS (11.6 versus 8.5 months respectively, HR = 0.53, *p* = 0.0005) but without benefit in OS (41 versus 42 months respectively, HR = 1.13, *p* = 0.70) [40]. Pazopanib appears to be a potential option after chemotherapy.

**Surufatinib** is a TKI targeting VEGFR, fibroblast growth factor receptor (FGFR) 1 and CSF1R, recently evaluated in a single-arm phase Ib/II study. Surufatinib resulted in an ORR of 19%, a DCR of 91% and a mPFS of 21.2 months for patients with panNETs [41].

**Bevacizumab** is a monoclonal antibody that targets VEGF. The non-randomized BETTER phase II trial tested the combination bevacizumab + 5-fluorouracil + streptozotocin in 34 patients with progressive, well-differentiated metastatic panNETs, with a DCR of 80%, a two-year mPFS and a two-year mOS of 88% [42]. However, there is not enough solid data on its efficacy in panNETs to recommend it.

Everolimus and sunitinib are approved for progressive G1/G2 panNETs, independent of Ki-67 and tumor load. Because of their low ORR but prolonged PFS and potential toxicity, they are generally used as a second-line after chemotherapy failure. Targeted therapies can be combined with SSAs in functional panNETs. In non-functional panNETs, this combination cannot be recommended in the absence of a study comparing the targeted drug with octreotide or lanreotide to the targeted drug alone. However, there may be a rationale. A phase II study showed a 92% DCR with everolimus in combination with octreotide in a first-line setting in gastro-entero-pancreatic (GEP) NET [43]. In contrast, a trial on progressive panNETs compared everolimus versus everolimus plus pasireotide, a new SSA, without any significant difference on PFS or OS [44].

### 2.4. Future Perspectives for Advanced/Metastatic PanNETs

Future perspectives for advanced/metastatic panNETs are summarized in Table 1. Strategy issues are raised with clinical trials comparing TEMCAP versus streptozotocin + 5-fluorouracil (NCT03351296) and TEMCAP versus capecitabine + dacarbazine (NCT03279601). The strategy of using the targeted therapy everolimus after treating with alkylating agents, versus before alkylating agents, is currently being evaluated (NCT02246127). Other targeted therapies such as cabozantinib (NCT03375320) or lenvatinib (NCT03950609) are also being evaluated. We can also note the place of trials on immunotherapy alone or in combination with certain targeted therapies.

## 3. Particular Metastatic Locations

### 3.1. Bone Metastases

Bone metastases occur in approximately 6–12% of patients with NENs and, along with liver metastases, are associated with a poor prognosis [45,46]. Their incidence may increase with the increasing use of imaging modalities with 68Ga-labeled tracers, which are particularly sensitive for bone metastases [47].

Bisphosphonates and denosumab may be used early in the management of bone metastases to delay the onset of bone pain. Bisphosphonates may also have the potential to improve OS [46]. Another valid option for the treatment of pain caused by bone metastases is palliative radiation, which has been shown to have a positive effect on quality-of-life [48]. Surgery, radiofrequency ablation and cryotherapy may also be considered. In addition to symptomatic treatment of bone metastases, these patients should be treated with a systemic treatment such as peptide receptor radionuclide therapy (PRRT), chemotherapy or targeted agents. Retrospective data suggest some cytoreduction of bone metastases with PRRT [49].

### 3.2. Peritoneal Metastases

Peritoneal metastases develop in approximately 17% of patients with GEP NETs, but are more prevalent in patients with ileal/appendicular than in those with panNETs [50,51].

Complete resection of peritoneal metastases should be considered when possible. Based on the limited data available, patients with complete cytoreductive surgery survive longer than patients with incomplete cytoreductive surgery (CRS) [51,52]. Patients should be selected and low-grade or slowly progressing tumors are those that may benefit most from CRS. In addition, CRS should be considered only if complete cytoreduction can be expected.

The combination of CRS and hyperthermal intraperitoneal chemotherapy (HIPEC) to treat NET-derived peritoneal metastases has not been studied prospectively. In a retrospective series on 41 patients with peritoneal metastases derived in majority from small-bowel NETs, 28 underwent complete CRS + HIPEC and 13 complete CRS alone, with similar two-year survival rates (81 versus 73%, respectively, *p* = 0.73) [53]. Therefore, we do not have enough data to conclude on the efficacy or otherwise of HIPEC, a technique with high morbidity.

## 4. High-Grade PanNEC

In 2017, the WHO grading system changed the classification, separating grade 3 NENs in well-differentiated NETs and poorly differentiated NECs.

### 4.1. G3 Poorly Differentiated PanNEC

Grade 3 poorly differentiated panNECs are rare in the gastrointestinal tract and account for less than 5% of GEP NENs, whereas they are common in the form of small cell carcinoma (SCLC) in the lung. Therefore, most of the proposed therapeutics are based on an analogy with the SCLC. NECs are characterized by poor differentiation, Ki-67 > 20% (usually >50%), high biological aggressiveness and are mainly diagnosed at a metastatic stage, with frequent tumor-related symptoms. Once the diagnosis is made, the start of chemotherapy is a relative emergency.

Based on their efficacy in metastatic SCLC, the doublet cisplatin + etoposide has been widely used in GEP NECs, with ORR in the most recent series of approximately 30% and a one-year mOS [28,54,55,56,57].

Carboplatin can replace cisplatin with etoposide, with less toxicity. The efficacy of carboplatin appears to be quite similar to that of cisplatin in a large retrospective study of 252 patients, with an OS of 11 months and an ORR of 30%, however without a face-to-face comparison [57].

Another option is to replace etoposide with irinotecan. A japanese phase III study in 154 patients with SCLC showed the superiority of irinotecan + cisplatin over etoposide + cisplatin (mOS: 12.8 versus 9.4 months, respectively, *p* = 0.002) [58]. Two western phase III studies subsequently failed to show the superiority of this regimen, also in SCLC [59,60]. A Japanese phase II study showed the feasibility of cisplatin-irinotecan on 18 patients with metastatic NETs, with a mOS of 11.4 months [61]. Several retrospective eastern studies have suggested that the irinotecan + cisplatin doublet is moderately effective and well-tolerated in metastatic panNECs [62,63,64,65].

Currently, there is no evidence to support the use of a triplet chemotherapy: a phase II trial tested the addition of paclitaxel to cisplatin-etoposide in 78 patients, with a high ORR (53%) but no benefit on OS compared with the standard cisplatin-etoposide regimen and with a greater toxicity [66].

After progression under first-line treatment, little data is available on second and third-line therapies. Small retrospective series have shown the feasibility of treatment with CAPOX or FOLFOX, with an ORR between 23% and 29% and a mOS of 10 months [29,67,68]. FOLFIRI regimen was evaluated in a retrospective study on 19 patients who progressed on etoposide-platinum [69]. The ORR was 31%, mPFS was four months and mOS was 18 months, compared to 6.8 months without treatment. Temozolomide, alone or in combination with capecitabine and bevacizumab was evaluated in 25 patients with poorly differentiated NECs, with 33% ORR and an OS of 22 months [70]. Conversely, in another study, temozolomide had no benefit on ORR and an OS of 3.5 to 4 months in 28 patients with NECs [71]. Resumption of cisplatin + etoposide therapy may also be considered in patients with good initial response and progress after a treatment break of at least three months, in the absence of cumulative toxicity.

### 4.2. High-Grade Well-Differentiated PanNET (G3 NETs)

Grade 3 well-differentiated panNETs appeared to have a lower response to platinum-based chemotherapy compared to panNECs but also better survival [57,72].

It has been suggested that patients with G3 NETs may benefit from medical treatments used in G2 NETs, such as temozolomide + capecitabine, but there are a lack of data from prospective studies. A recent retrospective study on 55 patients with G3 NETs showed that alkylating agents achieved the highest ORR (46%), regardless of Ki-67 [73].

Differentiation may be a more important parameter than the Ki-67 proliferation index in clinical practice [8,74]. The Ki-67 level in NECs is mainly above 60%, whereas Ki-67 level in G3 NETs usually ranges from 20% to 50%. A recent review proposed a Ki-67 threshold of 50–60% as a minimum level for proposing platinum-based chemotherapy [75]. Patients with G3 panNETs should probably be treated like G2 panNETs patients, except for Ki-67 between 50% and 60%. In the present issue, an article is dedicated to the management of G3 NETs.

### 4.3. Future Perspectives in PanNECs

Table 2 summarizes selected ongoing clinical trials on medical treatments in advanced/metastatic pNECs. The doublet cisplatin + etoposide is being challenged by TEMCAP (NCT02595424) and by other associations such as cisplatin + everolimus (NCT02695459) or cisplatin + irinotecan (NCT03963193). In a second-line setting, FOLFIRI is being challenged by the association TEMCAP (NCT03387592) and by the association FOLFIRI + bevacizumab (NCT02820857). Some targeted therapies such as everolimus are also being tested in second line (NCT02113800) or as a maintenance treatment (NCT02687958). Immunotherapy is also being evaluated, as monotherapy or in combination with chemotherapy or targeted therapy.

## 5. Mixed Neuroendocrine–Non-Neuroendocrine Neoplasm

In 2010, the WHO classification of tumors named tumors from the GEP tract with an exocrine and a neuroendocrine component accounting for at least a third or 30% of the tumor mass, mixed adeno-neuroendocrine carcinomas (MANECs) [76].

The ENETS guidelines for the management of MANECs are to follow the recommendations for G3 NECs [74], as the neuroendocrine component in MANECs is usually poorly differentiated and predominant, both in the primary tumors and in distant metastatic sites [77]. However, other authors suggest treating MANECs as adenocarcinomas from the same site of origin, when the adenocarcinoma component is prevalent and/or the least differentiated [78].

In 2017, the WHO classification of tumors of endocrine organs has renamed MANECs MiNENs, in order to incorporate some non-neuroendocrine histologies, with “non-gland-forming” variants such as squamous cell carcinoma or sarcoma and precancerous lesions such as adenomas. 

A retrospective analysis from five European institutions studied 69 patients with a diagnosis of MiNENs [79]. The choice of treatment regimen appeared to be based on the predominant or most aggressive histology. In some cases, platinum-based regimens used for adenocarcinomas were preferred despite a predominant or more aggressive neuroendocrine component. Chemotherapy in patients with unresectable MiNENs appeared to prolong PFS and OS versus BSC.

To make an accurate diagnosis of MiNEN, since it depends on a 30% threshold of neuroendocrine component, a GEP tract tumor with mixed histology and/or an unconventional behavior under standard treatment should be reviewed by a pathologist with expertise in NETs. 

## 6. Treatment Algorithm

Table 1 represents the therapeutic algorithm for locally advanced and surgically unresectable panNETs.

G1 or G2 panNETs that are stable or slowly progressive over several months, asymptomatic and with a Ki-67 of less than 5% to 10% can be treated in first line with SSAs, or followed up. The SSAs, lanreotide autogel 120 mg or octreotide LP 30 mg may be used, but only lanreotide has been studied in panNETs. At progression, SSAs may be introduced if simple follow-up was performed. If the patient was already on SSAs, it is possible to double the dose. In addition, depending on the rate of progression and on the localization of the metastases, locoregional therapy, chemotherapy or targeted therapy with everolimus or sunitinib may be considered.

G1 or G2 panNETs that have progressed over the last few months, or that are symptomatic, or that have a Ki-67 greater than 5% to 10%, must be treated with chemotherapy as a first-line treatment. This chemotherapy may be streptozotocin + 5-fluorouracil or streptozotocin + doxorubicin or TEMCAP. At progression, the patient may be treated with a targeted therapy, with a low cytoreduction rate but prolonged PFS. At progression, it will be possible to prescribe a second-line chemotherapy or to discuss PRRT at a multidisciplinary consultation meeting or to consider inclusion in a clinical trial.

Well-differentiated G3 NET treatment is similar to that of G2 NETs, with chemotherapy with streptozotocin + 5-fluorouracil or TEMCAP. At progression, they can be treated with FOLFOX or FOLFIRI-based chemotherapy.

G3 poorly differentiated panNECs treatment is a relative emergency and first-line treatment should be cisplatin + etoposide. Cisplatin can be replaced by carboplatin. At progression, they may be treated with FOLFOX or FOLFIRI or included in a clinical trial.

Table 3 presents all randomized clinical trials (phase III and randomized phase II) that were performed for PanNENs, with the agents and their doses and the outcomes and most common or severe adverse effects.

**Figure 2:**
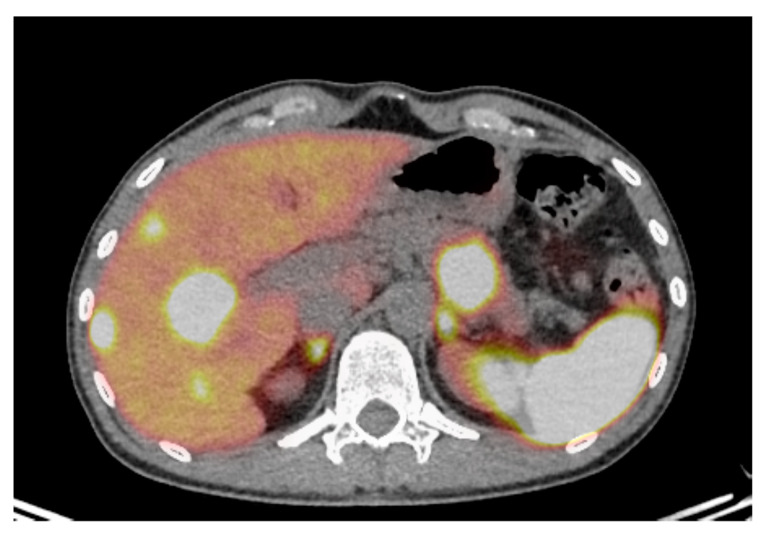
^68^Ga-DOTATOC transaxial fusion image in color scale of a 43-year-old patient with hepatic lesions identified as grade 2 (Ki 18%) neuroendocrine tumor. ^68^Ga-DOTATOC identified a pancreatic lesion and several hepatic metastases. Given its proliferation index, the patient was treated with doublet chemotherapy with temodal and capecitabine.

As an example, Figure 2 represents the ^68^Ga-DOTATOC of a 43-year-old patient with hepatic lesions identified as G2 NET with a Ki-67 of 18%. The functional imaging identified the pancreatic lesion and several hepatic metastases. Given its proliferation index, the patient was treated with TEMCAP.

## 7. Conclusions

Medical treatment remains the cornerstone for improving survival and preserving quality-of-life for advanced panNENs without the possibility of curative surgical treatment or locoregional or ablative therapy. The medical treatments available are varied: SSAs, alkylating agents, platinum agents, targeted therapies. Several elements have to be taken into consideration in order to make the right choice: the grade (G1/G2/G3), the Ki-67, the kinetics of evolution of the panNET and its symptomatic or non-symptomatic character. In summary, a G1 or G2, stable or slowly progressing panNET with a Ki-67 between 5% and 10%, in an asymptomatic patient, can be treated with SSAs. A progressive panNET, or one with Ki-67 over 5% to 10%, or a symptomatic patient should be treated with chemotherapy instead. This chemotherapy may be TEMCAP, or streptozotocin plus 5-fluorouracil, or streptozotocin plus doxorubicin. If they progress under chemotherapy, these patients can be treated with targeted therapy, a treatment that allows less cytoreduction and potential toxicity but prolonged PFS. Well-differentiated G3 panNETs are poorly studied but can be treated by analogy to G2 panNETs with alkylating agents, maybe except for Ki-67 between 50% and 60%. NECs should be promptly treated with cisplatin + etoposide as a first-line treatment. The scarcity and heterogeneity of NENs render their management difficult, thus all cases of NETs should be discussed at a specialized multidisciplinary tumor board and, if possible, reviewed by expert pathologists within the framework of specialized networks.

## Figures and Tables

**Table 1:** Selected ongoing clinical trials on medical treatments in advanced/metastatic pNETs.

Drug/Target	Phase	Population	Status	Recruiting Countries	NCT References
CAPDTIC vs. TEMCAP	IIR		Recruiting	China	NCT03279601
TEMCAP ± bevacizumab vs. LV5FU2-STZ ± bevacizumab (BETTER-2)	IIR	PD over the last 12 months	Recruiting	France	NCT03351296
Everolimus followed by STZ-5FU or STZ-5FU followed by everolimus	III	L1	Active, non recruting	Europe	NCT02246127
Cabozantinib vs. placebo	III	Progressive under everolimus	Recruiting	USA	NCT03375320
Lenvatinib + Everolimus	II	PD over the last 12 months	Recruiting	USA	NCT03950609
Abemaciclib (anti-CDK4 et 6)	II	>L1	Recruiting	USA	NCT03891784
Anlotinib + AK105	II	All lines	Not yet recruiting	China	NCT04207463
Entinostat (HDAC inhibitor)	II	Refractory	Recruiting	USA	NCT03211988
Tamoxifen	II	PD over the last 12 months, ER or PR+	Recruiting	USA	NCT04123262
Tamoxifen (HORMONET)	II	PD over the last 12 months, ER or PR+	Recruiting	Brazil	NCT03870399
Cabozantinib + Atezolizumab	II	Refractory	Recruiting	Spain	NCT04400474
Durvalumab + Tremelimumab (DUNE)	II	Progression to somatostatin analogs and one targeted therapy	Recruiting	Spain	NCT03095274
Cabozantinib + Nivolumab	II	PD over the last 12 months	Recruiting	USA	NCT04197310

Abbreviations: IIR: randomized phase II; CAPDTIC: capecitabine + deticene; CAPTEM: capecitabine/temodal; ER: estrogen receptor; L1: first-line therapy; L2: second-line therapy; PD: progressive disease; PR: progesterone receptor; STZ-5FU: streptozotocin + 5-fluorouracil; USA: United States of America vs: versus.

**Table 2:** Selected ongoing clinical trials on medical treatments in advanced/metastatic pNECs.

Drug/Target	Phase	Population	Status	Recruiting Countries	NCT References
Cisplatin + Etoposide vs. TEMCAP	IIR	>L1	Recruiting	USA	NCT02595424
Cisplatin + Everolimus	II	L1	Recruiting	Netherlands	NCT02695459
Cisplatin + Etoposide vs. Cisplatin + Irinotecan	II	L1	Not yet recruiting	China	NCT03963193
FOLFIRI vs. TEMCAP (SENECA)	IIR	L2	Recruiting	Italy	NCT03387592
Nanoliposomal Irinotecan With Fluorouracil and Leucovorin	II	Refractory	Recruiting	USA	NCT03736720
TAS-102 NEC: TAS-102	II	>L1	Recruiting	USA	NCT04042714
FOLFIRI + bevacizumab vs. FOLFIRI (BEVANEC)	IIR	L2	Recruiting	France	NCT02820857
Everolimus (EVINEC)	II	L2	Recruiting	Germany	NCT02113800
Everolimus as maintenance therapy vs. observation	IIR		Recruiting	Italy	NCT02687958
Pembrolizumab	II	>L1	Recruiting	USA	NCT03136055
Nivolumab ± Ipilimumab (NIPINEC)	IIR	L2	Recruiting	France	NCT03591731
Nivolumab + Ipilimumab	II	All lines	Recruiting	USA	NCT03420521
Platinum-doublet Chemotherapy and Nivolumab	II	L1	Recruiting	Spain	NCT03980925
Pembrolizumab with CT	II		Recruiting	USA	NCT03901378
Cabozantinib + Nivolumab + Ipilimumab	II	L2	Recruiting	USA	NCT04079712
Nivolumab and temozolomide	II	All lines	Recruiting	USA	NCT03728361

Abbreviations: IIR: randomized phase II; CT: chemotherapy; FOLFIRI: 5-fluorouracile + irinotecan; L1: first-line treatment; L2: second-line treatment; NEC: neuroendocrine carcinoma; TEMCAP: temozolomide/capecitabine; USA: United States of America; vs: versus.

**Table 3:** Randomized clinical trials (randomized phase II and phase III trials) in panNENs.

Trial	Number of Patients	Comparison Arms	Outcomes	Comparison	Adverse Effects
Caplin, NEJM, 2014 [7]	102	Lanreotide autogel 120 mg	PFS: not achieved	PFS: HR 0.47; *p* <0.001	Diarrhea
	102	Placebo	PFS: 18 months		
Moertel et al., NEJM, 1992 [12]	33	Streptozotocin 500 mg/m^2^ + 5-fluorouracil 400 mg/m^2^ for 5 days every 6 weeks	ORR: 45% TTP: 6.9 months OS: 1.4 years	ORR: *p* = 0.05 TTP: *p* = 0.001 OS: *p* = 0.004	Vomiting, hematological depression, renal insufficiency
	36	Streptozotocin 500 mg/m^2^ for 5 days every 6 weeks + doxorubicin 50 mg/m^2^ on days 1 and 22	ORR: 69% TTP: 20 months OS: 2.2 years		
	33	Chlorozotocin 150 mg/m^2^ every 7 weeks	ORR: 30% OS: 1.5 years		
Kunz, JCO, 2018 (abstract) [23]	72	Temodal 200 mg/m^2^ days 1–5	PFS: 14.4 months OS: 38.0 months	PFS: HR 0.58, *p* = 0.023 OS: HR 0.42, *p* = 0.012	
	62	Temodal 200 mg/m^2^ days 10–14 + Capecitabine 750 mg/m^2^ twice a day 1–14	PFS: 22.7 months OS: not reached		
Raymond, NEJM, 2011 [32]	86	Sunitinib 37.5 mg/day	PFS: 11.4 months ORR: 9.3%	PFS: HR 0.42, *p* > 0.001 ORR: *p* = 0.007	Diarrhea, vomiting, asthenia, neutropenia, hypertension, palmar-plantar erythrodysesthesia
	85	Placebo	PFS: 5.5 months ORR: 0%		
Yao, NEJM, 2011 [38,39]	207	Everolimus 10 mg/day	PFS: 11.0 months OS: 44 months	PFS: HR 0.42, *p* > 0.001 ORR: *p* = 0.007	Diarrhea, vomiting, asthenia, neutropenia, hypertension, palmar-plantar erythrodysesthesia
	203	Placebo	PFS: 4.6 months OS: 37.7 months		
Bergsland, JCO, 2019 (abstract) [40]	97	Pazopanib 800 mg/day	PFS: 11.6 months OS: 41 months	PFS: HR 0.42, *p* > 0.001 ORR: *p* = 0.007	Diarrhea, vomiting, asthenia, neutropenia, hypertension, palmar-plantar erythrodysesthesia
	74	Placebo	PFS: 8.5 months OS: 42 months		

Abbreviations: HR: hazard ratio; ORR: overall response rate; OS: overall survival; PFS: progression-free survival; TTP: time to progression.

## Abbreviations

CAPOX	Capecitabine + oxaliplatin
CRS	Cytoreductive surgery
CSFR	Colony-stimulating factor 1 receptor
DCR	Disease control rate
ENETS	European Neuroendocrine Tumor Society
FGFR	Fibroblast growth factor receptor
FOLFOX	5-fluorouracil + oxaliplatin
G1	Grade 1
G2	Grade 2
G3	Grade 3
GEP	Gastro-entero-pancreatic
HR	Hazard Ratio
HIPEC	Hyperthermal intraperitoneal chemotherapy
LAR	Long-Acting Release
MANECs	Mixed adeno-neuroendocrine carcinomas
MGMT	O6-methylguanine DNA methyltransferase
MiNENs	Mixed neuroendocrine non-neuroendocrine neoplasms
mOS	Median overall survival
mPFS	Median progression-free survival
mTORC1	Mammalian target of rapamycin complex 1
NANETS	North American NeuroEndocrine Tumors Society
NECs	Neuroendocrine carcinomas
NENs	Neuroendocrine neoplasms
NETs	Neuroendocrine tumors
ORR	Objective response rate
PanNENs	Pancreatic neuroendocrine neoplasms
PanNETs	Pancreatic neuroendocrine tumors
PDGFR	Platelet-derived growth factor receptor
PRRT	Peptide receptor radionuclide therapy
SCLC	Small cell carcinoma
SSA	Somatostatin analogs
SSTRs	Somatostatin receptors
TEMCAP	Temodal + capecitabine
TKI	Tyrosine kinase inhibitor
TTP	Time to Tumor Progression
VEGFR	Vascular endothelial growth factor receptor
WHO	World Health Organization
